# T157. FRONTOSTRIATAL CONNECTIVITY IN TREATMENT-RESISTANT SCHIZOPHRENIA: RELATIONSHIP TO POSITIVE SYMPTOMS AND COGNITIVE FLEXIBILITY

**DOI:** 10.1093/schbul/sby016.433

**Published:** 2018-04-01

**Authors:** Vanessa Cropley, Eleni Ganella, Cassandra Wannan, Andrew Zalesky, Tamsyn Van Rheenen, Chad Bousman, Ian Everall, Alexander Fornito, Christos Pantelis

**Affiliations:** 1 The University of Melbourne; 2 University of Calgary; 3 Institute of Psychiatry, Psychology & Neuroscience, King’s College London; 4 School of Psychology and Psychiatry & Monash Biomedical Imaging, Monash University; 5 Melbourne Neuropsychiatry Centre, University of Melbourne

## Abstract

**Background:**

The frontostriatal circuits linking different parts of the frontal cortex to subregions of the striatum are proposed to regulate different aspects of cognition, executive function, affect and reward processing. Dysregulation of these brain circuits is also known to be important in the etiology of psychotic disorders, with the magnitude of dysfunction correlating with the severity of positive symptoms. These observations suggest that the integrity of brain circuits connected to the striatum is important for antipsychotic treatment response as well as specific cognitive processes. However, not all individuals with schizophrenia benefit from antipsychotic treatment, with up to 20% of individuals considered to be treatment-resistant. These individuals also show pervasive impairments in cognition, including cognitive flexibility. Nevertheless, few studies have examined striatal connectivity in treatment-resistant schizophrenia (TRS), particularly in relation to positive symptomatology and specific cognitive deficits subserved by the striatal circuits. This study therefore aimed to (i) assess for disruptions in frontostriatal connectivity in a sample of TRS and (ii) assess the relationship between the frontostriatal circuits with positive symptoms and attentional set-shifting (cognitive flexibility) given recent associations with the dorsal striatal circuit.

**Methods:**

Resting-state functional magnetic resonance imaging was used to investigate functional connectivity (FC) in 42 TRS participants prescribed clozapine (30 males, mean age=41.3(10)), and 42 healthy controls (24 males, mean age=38.4(10)). The whole striatum (caudate, putamen and nucleus accumbens) and the left and right dorsal striatum were separately seeded as regions of interest, and Pearson’s correlations between the seeds and all other voxels comprising cortical and subcortical gray matter were investigated. For brain regions that showed significant group differences in FC with the striatal seeds, Pearson’s correlations explored the relationship between the strength of connectivity with positive symptoms and attentional set-shifting (extradimensional shift errors) as measured with the CANTAB intra-/extradimensional set shift task.

**Results:**

In comparison with healthy controls, TRS patients displayed significantly reduced FC between the whole striatum and the bilateral anterior cingulate, cerebellum, precuneus, right and left frontal pole and left insular/temporal pole, and reduced FC of the left and right dorsal striatum with cerebellum, and between the right dorsal striatum and bilateral cingulate and right frontal pole. Reduced FC between the whole striatum and precuneus and insular/temporal pole was associated with greater delusions of jealousy (p<.002 uncorrected); no other associations with positive symptoms were detected. In the entire sample, reduced FC from all striatal seeds was associated with greater extradimensional errors, indicating worse cognitive flexibility. These associations were not detected in TRS and controls separately.

**Discussion:**

Our preliminary findings reveal reduced striatal FC in TRS, including hypoconnectivity of the dorsal striatal circuit. In contrast to early psychosis, reduced dorsal striatal connectivity does not appear to mediate positive symptoms. Our finding relating hypoconnectivity of the striatal circuits with impaired cognitive flexibility is partly consistent with recent observations in other psychiatric disorders, although such deficits appear not specific to the dorsal circuit and to TRS. Future work will examine connectivity of the ventral striatum, as well as striatal connectivity in early-onset psychosis and siblings of patients with schizophrenia.

